# Stereotactic Navigation-Assisted Laparoscopic Resection of Challenging Low Pelvic Tumors: A Case Series

**DOI:** 10.3390/jcm13051233

**Published:** 2024-02-21

**Authors:** Guglielmo Niccolò Piozzi, Jung-Myun Kwak, Ji-Seon Kim, Se-Jin Baek, Jin Kim, Seon-Hahn Kim

**Affiliations:** 1 Division of Colon and Rectal Surgery, Department of Surgery, Korea University Anam Hospital, Korea University College of Medicine, Goryeodae-ro, Seongbuk-gu 73, Seoul 02841, Republic of Korea; guglielmopiozzi@gmail.com (G.N.P.); xezin@korea.ac.kr (S.-J.B.); mrgs@korea.ac.kr (J.K.); kimseonhahn@um.edu.my (S.-H.K.); 2 Department of Colorectal Surgery, Portsmouth Hospitals University NHS Trust, Southwick Hill Road, Portsmouth PO6 3LY, UK; 3 Department of Surgery, Faculty of Medicine, Universiti Malaya, Kuala Lumpur 50603, Malaysia

**Keywords:** stereotactic surgery, image-guided surgery, navigation, laparoscopy, pelvic surgery, rectal cancer

## Abstract

(1) **Introduction**: The laparoscopic approach to low pelvic tumors is challenging and hindered by suboptimal tumor visualization and dissection, with possible oncological failure. Stereotactic navigation provides real-time image guidance that may optimize safety, accuracy, and precision when dissecting challenging low pelvic tumors. (2) **Methods:** Preoperative CT images were acquired with eight skin-fixed fiducials and loaded into a navigation system. A patient tracker was mounted on the bed side. Patient-to-image paired point registration was performed, and an instrument tracker was mounted on a laparoscopic instrument and calibrated for instrument tracking. Surgical operations were performed with real-time stereotactic navigation assistance. (3) **Results**: Three patients underwent stereotactic navigation surgery. Fiducial registration errors were good to optimal (±1.9, ±3.4, and ±3.4 mm). Lesions were easily identified and targeted with real-time navigation. Surgeries were uneventful. Histopathology examinations identified one retro-rectal schwannoma, one lateral pelvic recurrence from rectal adenocarcinoma, and one advanced anal canal carcinoma. No navigation-related complications, readmissions, or postoperative mortalities were observed. (4) **Conclusions:** The application of laparoscopic stereotactic navigation surgery to complex low pelvic tumors is feasible and could impact oncological surgical quality by enabling tumor targeting and ensuring resection margins. Further wider series are needed to confirm stereotactic navigation’s impact on challenging low pelvic tumors.

## 1. Introduction

Stereotactic navigation surgery (SNS) is a well-established surgical strategy with wide applications in neurological, otolaryngological, and orthopedic surgery, during which surgical dissection is applied between the boundaries of a fixed bony space [1,2]. SNS is characterized by the integration of an intraoperative surgical view and preoperative images through the use of tracked surgical instruments for real-time assisted surgery [3]. SNS allows localization, targeting, and guiding of surgical procedures, especially when the target is not easily recognizable or occult. SNS can benefit the surgeon by enhancing surgical precision, reducing invasiveness, and providing theoretically fewer morbidities. Atallah et al. were the first to report the clinical use of SNS during colorectal surgery for an advanced rectal cancer abutting the prostate that was subjected to transanal minimally invasive surgery for total mesorectal excision [4]. Their report demonstrated that the bony pelvis, like the skull, can be an optimal field for SNS application. SNS was then successfully applied to a transanal total mesorectal excision (TaTME) to provide real-time position assessment during the transanal phase, improving the surgeon’s spatial awareness and accuracy, and the safety of the TaTME [3,5,6]. SNS was also applied to assist en bloc pelvic neoplastic resections [7,8] and laparoscopic lateral pelvic lymph node dissections (LPLND) [9].

SNS is expected to suit especially challenging cases [4], with the possibility to also be an aiding technology for the learning phase curve. SNS can benefit the surgical dissection of oncological R0 resections, and can aid surgeons by improving safety through vital structures recognition [4]. However, SNS is still limited in its adoption in colorectal surgery, with few reports showing a complex technique without providing possible clear indications and benefits for its use in colorectal surgery.

This study aimed to explore the role, feasibility, and efficacy of SNS in the surgical treatment of complex pelvic cases in a tertiary referral center with expertise in colorectal cancer surgery.

## 2. Materials and Methods

### 2.1. Study Population

Three patients underwent laparoscopic surgery with SNS between February 2019 and November 2020 in a tertiary referral center for colorectal cancer surgery at the Korea University Anam Hospital, Seoul, South Korea. This study was approved by the Institutional Review Board (#2022AN0084). All patients were informed about the study and agreed to undergo SNS. All surgeries were performed by two surgeons (J.-M.K. and J.K.).

### 2.2. Rationale

Real-time navigation during SNS requires five elements: (1) preoperative high-quality images (intraoperative if possible) performed with radio-opaque markers (skin-fixed fiducials), (2) a fixed-point patient tracker, (3) an instrumental tracker, (4) an infrared ceiling camera in the direct line-of-sight of both the patient and instrumental trackers, and (5) navigational software. Once the system is set, real-space (patient) coordinates are associated with digital-space coordinates (radiologic images) to generate real-time navigation on a multiplanar format on the three-axis.

### 2.3. Image Acquisition

**Figure 1:**
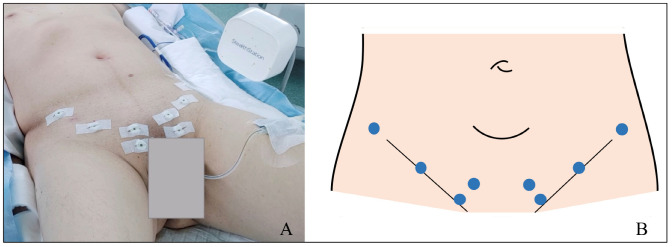
Skin-fixed fiducial placement positions: (**A**) operative view and (**B**) schematic view.

After tumor staging and acquiring the patient’s agreement to undergo SNS, a preoperative computed tomography (CT) scan with 1 mm slice thickness was performed the day before surgery, with the patient laying in the supine position. Before image acquisition, eight skin-fixed fiducials were applied to the lower abdomen skin at the level of the anterior superior iliac spine (*n* = 2), inguinal ligament (*n* = 2), and pubic bone (*n* = 4) (Figure 1A,B). These radio-opaque markers are important for the registration process that associates real-space coordinates with digital-space coordinates. These markers must be placed near the target area being navigated and on fixed positions not influenced by ventilation, heartbeat, or abdominal cavity insufflation. For this reason, bony and ligamentous structures are optimal.

### 2.4. Operating Theatre Setup

**Figure 2:**
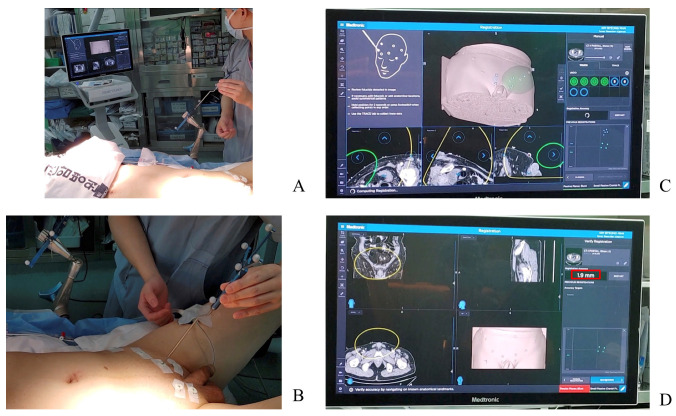
Intraoperative steps for patient-to-image paired point registration: (**A**) bed rail-mounted patient tracker registration, (**B**) skin-fixed fiducials registration, (**C**) software view during skin-fixed fiducials registration, and (**D**) software view after registration showing the fiducial registration error (red box).

A StealthStation™ S8 surgical navigation system (Medtronic, Inc., Minneapolis, MN, USA) with cranial setting software was used during this study as a navigation platform. Preoperative CT images for stereotactic navigation guidance surgery were loaded into the system and verified to meet the system’s minimum requirements. General anesthesia was induced, and the patient was positioned in the lithotomy position with the legs slightly flexed. The patient tracker was mounted to the bed rail on the patient’s left side and registered with a registration pointer (Figure 2A). A registration pointer was then used to touch each skin-fixed fiducial to complete the patient-to-image paired point registration. This allowed the system to correlate all the CT scan’s digital points to corresponding points on the patient. The system’s accuracy was checked by calculating the fiducial registration error, which should be below 5 mm, and patient registration was performed (Figure 2B–D). Next, the instrument tracker was mounted on the distal shaft of a laparoscopic instrument and calibrated so that the tracker could provide real-time information regarding the device’s tip position in the CT image space. Both the patient and instrument tracker were always kept within the line-of-sight of the ceiling-mounted receiver. The navigation software received constant information from both trackers. The patient tracker was maintained in a fixed position, along with all the surgery, while the instrument-tracker was moved according to procedure requirements, taking care not to change its position relative to the operating tip of the device. At this point, the SNS was started.

## 3. Results

### 3.1. Case 1: Retro-Rectal Tumor

**Figure 3:**
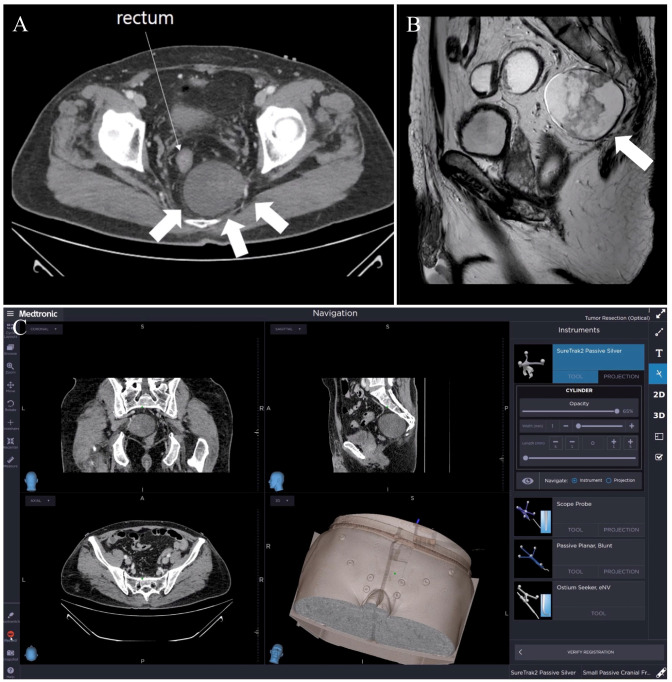
CT scan showing the retro-rectal tumor (thick arrow): (**A**) axial view and (**B**) sagittal view. (**C**) Multiplanar real-time view on the navigation software interface of the dissection above the tumor. The blue dot in each quadrant shows the instrument’s tip position for each plane.

An incidental 6.6 cm low attenuated mass was found in the left retro-rectal space abutting the left sacral nerve during a CT scan of a 63-year-old man with clinical suspicion of acute appendicitis (Figure 3A,B). A neurogenic tumor was suspected. After setting the stereotactic navigation system, a fiducial registration error of ±1.9 mm was reported. A five-trocar laparoscopic setting was used. After completing postero-lateral mobilization, the rectum was anteriorly retracted and the tumor was searched, identified, and targeted using the tracked instrument (laparoscopic scissor) (Figure 3C). The tumor was carefully dissected, preserving surface integrity, and avoiding presacral bleeding. The surgery’s duration was 235 min. The estimated blood loss was 100 mL. No navigation-related complication was observed. The pathological report revealed a 6.5 × 2.4 cm benign schwannoma. The postoperative course was uneventful, and the patient was discharged on postoperative day 7. Follow-up was uneventful at 14 months. SNS specifically contributed to the identification and dissection of this bulky retro-rectal tumor.

### 3.2. Case 2: Rectal Cancer Local Recurrence

**Figure 4:**
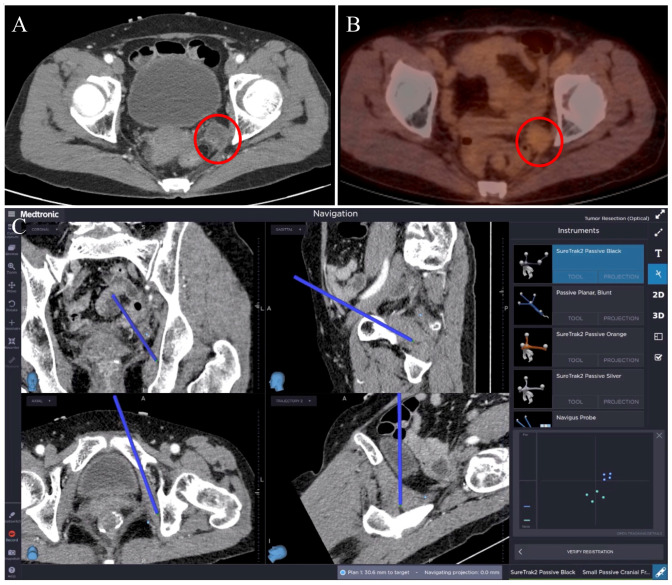
(**A**) CT scan and (**B**) positron emission tomography showing a pelvic left lateral lesion (red circles). (**C**) Multiplanar real-time view on the navigation software interface of the dissection along the lateral pelvic wall. The blue straight line in each quadrant shows the instrument’s position for each plane at the inferior margin of the pelvic left lateral recurrence.

A 60-year-old woman developed a 2.2 cm pelvic lateral recurrence at the left internal iliac chain 33 months after neoadjuvant chemoradiotherapy and robotic low anterior resection for a ypT3 N2b(9/19) M0 mid rectal tumor with a positive circumferential resection margin (Figure 4A,B). The patient was also submitted to 12 cycles of FOLFOX (leucovorin, fluorouracil, and oxaliplatin). The recurrence showed mild hypermetabolism in a positron emission tomography (PET/CT) scan. After setting the stereotactic navigation system, a fiducial registration error of ±3.4 mm was reported. A laparoscopic extra-peritoneal dissection was performed with three trocars (one at the umbilicus and one at each of the left and right low abdominal quadrants). The tumor was searched, identified, and targeted using the tracked instrument (laparoscopic bowel grasper). The dissection proceeded from the lateral margin to the inferior margin and was then finalized. The navigation system was constantly used during the dissection (Figure 4C). The pathologic report revealed a metastatic mucinous type of adenocarcinoma. The surgery’s duration was 133 min. The estimated blood loss was 50 mL. No navigation-related complications were observed. A postoperative CT scan confirmed the complete removal of the lateral recurrence. The postoperative course was uneventful, and the patient was discharged on postoperative day seven. The patient was then submitted to FOLFIRI (leucovorin, fluorouracil, and irinotecan) and bevacizumab, and was alive at a follow-up 36 months after the recurrence. SNS especially contributed during dissection in the extra-peritoneal space.

### 3.3. Case 3: Advanced Anal Cancer

**Figure 5:**
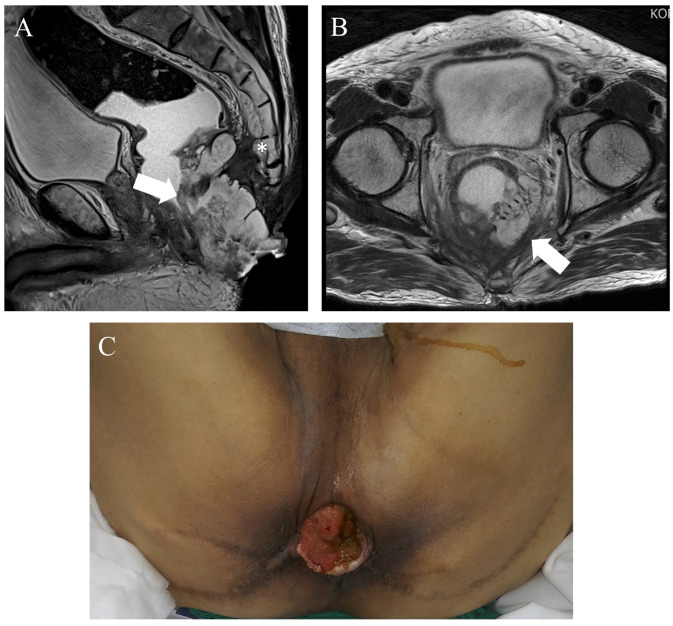
(**A**,**B**) CT scans and intraoperative view (**C**) of an extensive anal cancer (arrows) with infiltration of the presacral area (white star).

**Figure 6:**
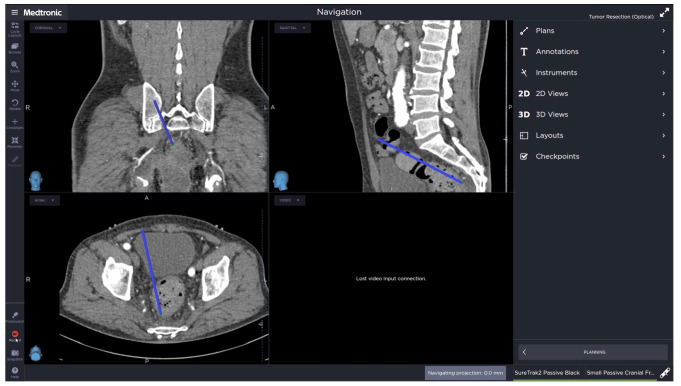
Multiplanar real-time view of the pelvic dissection on the navigation software interface. The blue straight line in each quadrant shows the instrument’s position relative to S4, which was the landmark for the transabdominal dissection before the perineal phase.

A 69-year-old male was diagnosed with anal cancer. After a wide excision evidenced a mucinous carcinoma, local radiotherapy (34 fractions, total 6120cGy) was applied. An abdominoperineal resection was proposed, but refused by the patient, who was lost in follow up. The patient returned for care 68 months after radiotherapy with an advanced anal canal cancer infiltrating the presacral area (Figure 5). After multidisciplinary tumor evaluation, a laparoscopic abdominoperineal resection (APR) with an S4 sacrectomy was planned. For this patient, the navigation system was adopted to intraoperatively identify the level of S4 to optimize the pelvic dissection for safe dissection. After setting the SNS, a registration error of ±3.4 mm was reported (Figure 6). The pelvic dissection was interrupted at S4. Following this, a perineal phase for extralevator APR was performed, although the initial plan was an S4 sacrectomy. A perineal reconstruction with pedicled vertical rectus abdominis musculocutaneous flap coverage was performed by the plastic surgeon. The surgery’s duration was 622 min. The estimated blood loss was 150 mL. No navigation-related complications were observed. The pathological report revealed a rpT4a N0(0/24) mucinous adenocarcinoma with a positive circumferential resection margin and a 0.8 cm distal resection margin. The postoperative course was complicated by a perineal wound infection, which required several surgical revisions with debridement by the plastic surgeon. The patient was discharged on postoperative day 158. The patient was alive at 54 month follow-up without any evidence of recurrence.

## 4. Discussion

Despite SNS originally being described in 1986, it has only recently been applied during colorectal surgery, and has a promising role in this surgical field [4]. Preoperative and intraoperative procedures for SNS may be time-consuming, and require a specific platform and advanced training, which make it unfit for routine colorectal surgery. However, SNS can provide potential aid to the surgeon in challenging cases, such as tumors outside the boundaries of the mesorectal fascia (e.g., retro-rectal tumors) and pelvic recurrences, and in guiding dissections in unfamiliar spaces (e.g., LPLND). Dissection often relies on palpation or visual inspection, which can be challenging in the deep pelvis, increasing the likelihood of approximating tumor boundaries and being responsible for inadequate tumor margins or tumor disruption. SNS can increase the surgeon’s spatial awareness in such difficult cases, and thus help avoid the risk of injuring surrounding structures (as in Case 3).

With the rapid spread of minimally invasive surgery, LPLND is increasingly laparoscopically or robotically performed. However, LPLND is a technically demanding procedure due to anatomical complexity and variations [10]. On the other hand, large vessels in the lateral pelvic wall are fixed; therefore, they are good landmarks for positional information. Based on this information, SNS can assist surgeons in making operational decisions by displaying positions of surgical tools and locations of anatomical landmarks on a monitor [9,11]. For complete lateral lymph node dissection or removal of single malignant nodes, navigation technology could be helpful in localizing suspect lymph nodes and decreasing operating time, minimizing the extent of dissection of the pelvic sidewall, and preventing damage to vital surrounding structures [8].

The complete resection of local recurrences of rectal cancer is challenging because of complex anatomical changes following primary surgery and indefinite cancer demarcation. Hojo et al. [12] reported R0 resections in 8 of 11 locally recurrent colorectal cancer cases. They mentioned that 3D navigation tools are potentially useful in completing the resection of intra-abdominal recurrence of colorectal cancer. A recently reported NAVI-LARRC prospective study evaluated the feasibility of SNS in a patient with locally advanced primary and recurrent rectal cancer. R0 resection was obtained in 6/8 (75%) locally advanced rectal cancer cases and 6/9 (69%) locally advanced recurrent rectal cancer cases. The authors concluded that selected patients are likely to benefit from navigation-assisted surgery [13].

The present case series expanded the role of SNS in pelvic surgery outside its application in TaTME [3,6] by providing three different surgical scenarios for which this procedure could be beneficial. However, despite reported optimal margins in Cases 1 and 2 and the safe dissection in Case 3, it is not possible at this stage to demonstrate that SNS was the main contributing factor in these surgical successes. A randomized controlled trial demonstrating the superiority of SNS over standard surgery is required, but may not be clinically feasible for such rare, selected, and heterogeneous cases. Therefore, wider series are needed to confirm the role of SNS in low pelvic tumor treatment and to further define indications for this innovative technology, which is still not standardized or used in ordinary clinical practice.

Kok et al. [14] hypothesized that using SNS during advanced and recurrent rectal cancer resection would enable the surgeon to utilize preoperative imaging during the surgical procedure and improve the surgical outcome by yielding a higher complete resection rate when compared to a historical cohort’s results. They compared the complete resection rate of 33 patients (14 with primary tumors with threatened CRMs and 19 with recurrent rectal cancer) with results of a historical cohort of 142 patients (101 with primary tumors and 41 with recurrent rectal cancer). A significant difference in complete resection rates was found between the navigation and historical cohorts after recurrent rectal cancer resection (78.9% vs. 48.8%, respectively; *p* = 0.047). For locally advanced primary tumor resection, the difference was not significant (92.9% vs. 84.2%, respectively; *p* = 0.69). As a result, Kok et al. have demonstrated that SNS offers a potential advantage to patients who are undergoing attempted major curative extirpative procedures for recurrent rectal cancer.

Our SNS strategy was similar to that applied by Atallah et al. [4,5,6,7,15] and Kwak et al. [3]; however, our strategy may be technically easier. First, we adopted a reduced number of only eight skin-fixed fiducials as a more practical strategy, which still allowed for a good fiducial registration error that was similar to those of previous reports. Second, when performing rectal resections in our center, patients were usually positioned in the lithotomy position with the legs slightly flexed (nearly straight); therefore, compared to our previous report [3] there was no need to adopt a forced sacral tilt by placing a ten-degree wedge under the patient’s sacrum during the preoperative CT scan to mimic the pelvic organ movement caused by the lithotomy position. This makes our approach easier to apply, which is crucial for increasing the applicability of SNS. The present case series, with its good fiducial registration errors, also confirmed the feasibility of using a bed-fixed patient tracker with no need for previous invasive approaches, such as fixing it on the patient’s anterior superior iliac spine by drilling a rod [3]. However, it is important to secure the patient’s position with a bean bag to prevent the patient from sliding due to table tilt. Finally, the absence of signal dropout during these three cases, even during long procedures (as for Case 3), showed the reliability of SNS in this setting.

Atallah et al. were the first to integrate SNS into robotic surgery, further developing this approach into the integrated digital surgery revolution [5]. The robotic approach could gain an advantage from SNS, especially because of its lack of haptic feedback, which reduces the possibility of palpation identification. Also, the robotic platform allows for image integration inside the surgeon’s console for better direct control of navigation.

This study had limitations, as it provided a small case series of patients with surgical scenarios that may have benefited from SNS. Moreover, this study was performed in a tertiary oncological center with expertise in minimally invasive surgery, which could cause non-generalizability of results. However, this study provided three potential scenarios in which SNS could be implemented in advanced colorectal centers to optimize surgical outcomes. Future studies evaluating the role of robotic platforms with augmented reality technologies could provide the additional technological footprints required to make SNS access easier in surgical theatres.

## 5. Conclusions

SNS is feasible for targeting challenging low pelvic tumors where it could provide significant aid for oncologically safe dissections. SNS safety should be confirmed in wider series. SNS may have a role in tertiary referral centers in cases that would be troublesome with standard surgery. Further extended series are needed to confirm the feasibility of SNS, explore its cost-effectiveness, further standardize its methodology, and define its specific indications in pelvic surgery.

## Data Availability

The dataset used or analysed during the current study is available from the corresponding author upon reasonable request.
